# Common, germline genetic variations in the novel tumor suppressor *BAP1* and risk of developing different types of cancer

**DOI:** 10.18632/oncotarget.20465

**Published:** 2017-08-24

**Authors:** Moubin Lin, Liren Zhang, Michelle A.T. Hildebrandt, Maosheng Huang, Xifeng Wu, Yuanqing Ye

**Affiliations:** ^1^ Department of General Surgery, Yangpu Hospital, Tongji University School of Medicine, Shanghai, People’s Republic of China; ^2^ Department of Epidemiology, The University of Texas MD Anderson Cancer Center, Houston, Texas, USA

**Keywords:** BAP1, SNP, cancer risk

## Abstract

BRCA1 associated protein-1 (BAP1) is a novel tumor suppressor that has recently been shown to be somatically mutated in several cancers. The *BAP1* gene also carries rare germline mutations in families with a high incidence of several types of cancers, such as mesothelioma, uveal melanoma, lung adenocarcinoma, melanocytic neoplasms, and renal cell carcinoma. To test the hypothesis that common, germline genetic variants in *BAP1* may also contribute to the risk of developing different types of cancer, we genotyped germline single nucleotide polymorphisms (SNPs) for *BAP1* in a large population of patients with cancer, including 2,340 with colorectal cancer, 1,436 with bladder cancer, 3,313 with lung cancer, 1,325 with renal cell carcinoma, and 1,162 with esophageal cancer. We identified significant association of rs11708581 (P = 0.0034) and rs390802 (P = 0.015) with risk of renal cell carcinoma and rs12163565 (P = 0.038) with risk of lung cancer. Expression quantitative trait loci analysis in renal cell carcinoma using publicly available data from TCGA showed that the proxy SNPs for rs11708581 and rs390802 were negatively associated with the expression level of BAP1. Our study indicate that common germline genetic variants of *BAP1* play a role in mediating the risk of developing renal cell carcinoma and lung cancer.

## INTRODUCTION

BRCA1-associated protein 1 (BAP1) is a nuclear ubiquitin carboxy-terminal hydrolase or deubiquitinating enzyme (DUB) that play a role in regulating several cellular functions, such as cell cycle, differentiation, DNA damage response, and cell proliferation (reviewed by Carbone et.al. [1]). BAP1 was originally discovered as a binding partner for BRCA1 [2] and shown to inhibit cancer cell growth. Later studies showed that BAP1 was not responsible for deubiquitylation of BRAC1/BARD1 [3] and demonstrated that BAP1’s effect on cell growth was independent of BRCA1 [4]. BAP1’s role as a tumor suppressor has been supported by an *in vivo* tumorigenicity study, in whch BAP1 activity and nuclear localization restricted the growth of lung cancer cell lines [4]. That study also showed that BAP1 slows cell growth through altered G1-S checkpoint regulation. These functions in the regulation of the cell cycle, cell growth, and proliferation are mediated through interactions with HCF-1 (host cell factor-1) [5] and transcription factor YY1 (Ying Yang 1) [6], which result in transcriptional control of genes involved in these processes.

Recently, several studies have identified somatic and germline BAP1 mutations in a wide-range of cancers, including mesothelioma [7–9], uveal melanoma [10–12], lung adenocarcinoma [11], renal cell carcinoma [13, 14], meningioma [11], and melanocytic neoplasms [15]. These studies have shown that rare genetic alterations in BAP1 can be drivers of the tumorigenetic process in these tumors.

Lung cancer, colorectal cancer, bladder cancer, esophageal cancer, and renal cell carcinoma comprise an estimated 31.7% of the total cancers diagnosed this year in the United States and up to 43.8% of predicted cancer deaths [16]. The pathogenesis of these cancers is multi-factorial with a combination of both exposure and lifestyle factors (i.e. age, sex, co-morbidities, obesity, and occupation) as well as a genetic component. Large scale genome-wide association studies (GWASs) have been conducted for each cancer site and have identified a number of novel susceptibility loci [17–30]. However, the effects of known susceptibility loci do not account for the entire genetic component of these cancers, according to estimates of heritability [31]. Therefore, there is a need to identify new genetic factors that contribute to cancer risk as a step towards enhanced risk prediction.

Although these previous studies have provided the functional characterization of BAP1 and identified rare mutations in several cancers, the role of common germline genetic variants in *BAP1* on the risk of developing different types of cancer remains largely unknown. A recent large scale meta-analysis showed that common genetic variants in *APC* and *MLH1* genes, two genes known to have high-penetrance mutations contributing to familial colorectal cancer, had strong cumulative epidemiological evidence for a significant association with colorectal cancer risk [32]. In this study, we used a candidate gene based approach, which has been shown to play an important role in identifying additional genetic variants associated with a disease of interest even after the advent of GWAs [33]. We hypothesized that common, germline genetic variants in *BAP1* may also contribute to the risk of developing different types of cancer. To ensure a comprehensive coverage of common genetic variants in the *BAP1* gene, we used the tagging single nucleotide polymorphism (SNP) approach with an r^2^ of 0.80 and a minor allele frequency of >5% for SNP selection and further supplemented with two additional SNPs based on the basis of putative function. We performed this analysis in a large, multi-cancer site, case-control study comprised of 9,576 cancer cases (bladder cancer, lung cancer, renal cell carcinoma, esophageal cancer, and colorectal cancer) and a pool of 4,945 controls.

## RESULTS

### Host characteristics

This multi-cancer site analysis included a total of 9,576 cancer cases and 4,945 healthy controls (Table 1 and Supplementary Table 1). All patients included in the analysis were white. The number of cancer cases by type was as follows: bladder cancer, 1,436; colorectal cancer, 2,340; esophageal cancer, 1,162; renal cell carcinoma, 1,325; and lung cancer, 3,313.

**Table 1:** Study populations

Variables	Cases, N(%)	Controls, N(%)	P-value
**Bladder Cancer**			
**Total**	**1436**	**1436**	
**Age, mean(SD)**	65.40(11.09)	65.40(11.07)	1.00
**Sex**			1.00
Male	1147(80)	1147(80)	
Female	289(20)	289(20)	
**Smoking status**			<0.001
Never	340(28)	491(34)	
Ever	877(72)	941(66)	
**Colorectal Cancer**			
**Total**	**2340**	**2340**	
**Age, mean(SD)**	56.48(11.89)	56.52(11.83)	0.91
**Sex**			1.00
Male	1350(58)	1350(58)	
Female	990(42)	990(42)	
**Smoking status**			<0.001
Never	1270(54)	899(39)	
Ever	1070(46)	1432(61)	
**Esophageal Cancer**			
**Total**	**1162**	**1162**	
**Age, mean(SD)**	62.67(10.76)	62.68(10.74)	0.99
**Sex**			1.00
Male	1027(88)	1027(88)	
Female	135(12)	135(12)	
**Smoking status**			<0.001
Never	270(27)	398(34)	
Ever	718(73)	760(66)	
**Renal Cell Carcinoma**			
**Total**	**1325**	**1325**	
**Age, mean(SD)**	59.54(10.80)	59.54(10.78)	1.00
**Sex**			1.00
Male	882(66.57)	882(67)	
Female	443(33.43)	443(33)	
**Smoking status**			<0.001
Never	580(46)	488(37)	
Ever	687(54)	834(63)	
**Lung Cancer**			
**Total**	**3313**	**3313**	
**Age, mean(SD)**	62.40(10.81)	62.32(10.78)	0.78
**Sex**			1.00
Male	1824(55)	1824(55)	
Female	1489(45)	1489(45)	
**Smoking status**			1.00
Never	589(18)	589(18)	
Ever	2724(82)	2724(82)	

*SD–standard deviation. Number for each variable may not add up to total due to missing data.

### *BAP1* genetic variants and risk of developing different types of cancer

Overall, three *BAP1* genetic variants were significant, two with the risk of renal cell carcinoma and one with the risk of lung cancer (Table 2). The most significant association was observed for rs11708581. A 26% decrease in the risk of developing renal cell carcinoma (95% confidence interval [CI]= 0.61-0.91, P=0.0034) was observed for those carrying either the heterozygous or homozygous variant genotype for rs11708581. Significant reduced risk of renal cell carcinoma was observed for those carrying variant alleles of rs390802 (odds ratio [OR]=0.80, 95% CI= 0.67-0.96, P=0.015). Individuals carrying variant alleles of rs12163565 had a significantly increased risk of developing lung cancer (OR=1.11, 95% CI= 1.01-1.24, P=0.038) as well as an increased risk of developing bladder cancer (OR=1.17, 95% CI=0.99-1.39, P=0.070), although that increased risk was not statistically significant. However, none of the variants were significantly associated with the risk of esophageal cancer and colorectal cancer and the similar results were observed for esophageal cancer with the additional adjustment for alcohol consumption (data not shown). The association for rs11708581 with renal cell carcinoma remained significant even after adjustment for multiple comparison using the Bonferroni correction of 0.01 (=0.05/5 tests) while none of the other associations remained significant after Bonferroni correction.

**Table 2:** Effects of *BAP1* genetic variation on the risk of developing different types of cancer

SNP	Cases, N (cc/cv/vv)	Control, N (cc/cv/vv)	Model	*OR(95% CI)	p-value
**Bladder Cancer**					
rs11708581	1066\310\16	1064\325\23	Dom	0.90(0.74-1.10)	0.30
rs123598	1260\135\1	1272\133\2	Dom	1.04(0.79-1.36)	0.79
rs12163565	893\461\50	945\422\53	Dom	1.17(0.99-1.39)	0.070
rs390802	922\427\40	941\422\46	Dom	0.99(0.83-1.18)	0.89
rs13094687	617\632\135	613\631\163	Dom	1.06(0.89-1.26)	0.51
**Esophageal Cancer**					
rs11708581	880\247\20	848\261\20	Dom	0.94(0.76-1.16)	0.54
rs123598	1033\114\2	1046\95\2	Dom	1.07(0.79-1.45)	0.67
rs12163565	755\340\33	759\345\39	Dom	1.00(0.83-1.21)	1.00
rs390802	772\333\37	758\344\33	Dom	0.93(0.77-1.13)	0.49
rs13094687	502\488\135	516\484\142	Dom	1.00(0.88-1.13)	0.96
**Renal Cell Carcinoma**					
rs11708581	1028\251\9	982\295\18	Dom	0.74(0.61-0.91)	**0.0034**
rs123598	1173\127\1	1169\123\3	Dom	0.99(0.71-1.38)	0.95
rs12163565	825\420\56	861\391\55	Dom	1.05(0.86-1.29)	0.62
rs390802	918\340\34	861\400\35	Dom	0.80(0.67-0.96)	**0.015**
rs13094687	571\584\139	613\547\136	Dom	1.09(0.93-1.28)	0.30
**Lung Cancer**					
rs11708581	2521\685\41	2467\721\42	Dom	0.93(0.82-1.04)	0.21
rs123598	2937\320\3	2900\330\6	Dom	0.93(0.79-1.10)	0.39
rs12163565	2060\1073\135	2122\1001\129	Dom	1.11(1.01-1.24)	**0.038**
rs390802	2207\970\76	2163\970\94	Dom	0.96(0.86-1.06)	0.41
rs13094687	1476\1435\342	1468\1404\357	Dom	1.00(0.90-1.11)	0.99
**Colorectal Cancer**					
rs11708581	1767\503\30	1744\514\29	Dom	1.00(0.86-1.17)	0.80
rs123598	2083\224\1	2056\234\2	Dom	0.89(0.72-1.09)	0.93
rs12163565	1506\729\89	1501\700\95	Dom	1.07(0.94-1.22)	0.30
rs390802	1553\690\61	1526\697\64	Dom	0.97(0.85-1.11)	0.73
rs13094687	1062\1024\230	1054\992\241	Dom	1.00(0.88-1.13)	0.96

* adjusted for age, sex, and smoking status.

Dom – dominant,

c – common allele, v – variant allele.

### Stratified analysis for *BAP1* genetic variants and risk of of developing different types of cancer

To determine whether the effects of these significant variants were modified by epidemiological factors, we performed stratified analyses by age, smoking status, and sex (Table 3). The protective effects of both rs11708581 and rs390802 against renal cell carcinoma remained significant among male subjects, ever smokers and subjects younger than 65 yrs. The effect of rs12163565 with the risk of lung cancer remained significant among male and ever smokers. However, none of the interactions between these three SNPs and the stratification variables was significant.

**Table 3:** Effect of *BAP1* genetic variants by sex, smoking status, and age

SNP	Group	Case N(cc/cv/vv)	Control N (cc/cv/vv)	Model	*OR(95% CI)	P-value
**Sex**						
**Renal Cell Carcinoma**						
rs11708581	Male	689\165\5	652\197\16	Dom	0.75(0.60-0.95)	**0.017**
	Female	339\86\4	330\98\2	Dom	0.88(0.63-1.23)	0.45
rs390802	Male	625\217\22	575\263\26	Dom	0.77(0.63-0.95)	**0.014**
	Female	293\123\12	286\137\9	Dom	0.93(0.69-1.25)	0.65
**Lung**						
rs12163565	Male	1128\594\73	1203\531\59	Dom	1.19(1.04-1.37)	**0.013**
	Female	932\479\62	919\470\70	Dom	0.99(0.85-1.15)	0.90
**Smoking Status**						
**Renal Cell Carcinoma**						
rs11708581	Never	447\109\6	372\103\6	Dom	0.87(0.65-1.17)	0.36
	Ever	538\128\3	607\192\12	Dom	0.73(0.57-0.94)	**0.014**
rs390802	Never	390\159\14	329\140\11	Dom	0.96(0.74-1.25)	0.78
	Ever	485\168\18	529\260\24	Dom	0.73(0.58-0.91)	**0.0051**
**Lung**						
rs12163565	Never	377\187\18	375\177\29	Dom	1.00(0.78-1.27)	0.98
	Ever	1683\886\117	1747\824\100	Dom	1.12(1.00-1.26)	**0.047**
**Age**						
**Renal Cell Carcinoma**						
rs11708581	Subjects<65	679\157\5	639\196\15	Dom	0.72(0.57-0.91)	**0.0065**
	Subjects≥65	349\94\4	343\99\3	Dom	0.92(0.66-1.27)	0.60
rs390802	Subjects<65	601\226\18	552\265\32	Dom	0.77(0.62-0.95)	**0.014**
	Subjects≥65	317\114\16	309\135\3	Dom	0.90(0.67-1.21)	0.47
**Lung**						
rs12163565	Subjects<65	1103\584\72	1138\554\72	Dom	1.07(0.93-1.23)	0.32
	Subjects≥65	956\489\63	984\446\57	Dom	1.13(0.97-1.31)	0.12

*adjusted for age, and smoking status in sex stratification; adjusted for age and sex in smoking status stratification.

Dom - dominant.

c – common allele, v – variant allele.

### eQTL analysis

Because rs11708581 and rs390802 were not genotyped by The Cancer Genome Atlas (TCGA), we used 4 proxy SNPs for rs11708581 and 3 proxy SNPs for rs390802 (Table 4) to assess the association of these SNPs with expression level of BAP1. We found that BAP1 expression was negatively associated with two proxy SNPs for rs11708581 (rs17052053: r^2^=0.9, P value=0.006 and rs11713914: r^2^=1.0, P value=0.05) and one proxy SNP for rs390802 (rs11714402: r^2^=0.98, P value=0.023). The association of rs17052053 with *BAP1* expression remained significant after Bonferroni correction of 0.007 (=0.05/7 tests).

**Table 4:** eQTL analysis on the effect of Proxy SNPs on BAP1 expression level for renal cell carcinoma using data downloaded from TCGA

Proxy SNP	r^2^	SNP of interest	Coefficient	Standard error	P-value
rs1769	0.94	rs11708581	-0.062	0.042	0.142
rs6793317	0.9	rs11708581	-0.079	0.040	0.051
rs17052053	0.9	rs11708581	-0.113	0.041	**0.006**
rs11713914	1.0	rs11708581	-0.084	0.042	**0.050**
rs6763882	0.93	rs390802	-0.070	0.038	0.067
rs11714402	0.98	rs390802	-0.086	0.038	**0.023**
rs123602	0.98	rs390802	-0.050	0.041	0.222

## DISCUSSION

*BAP1* has generated much attention owing to the consistent findings of rare germline or somatic mutations in a wide-range of cancer sites [7–15]. However, the role of common germline genetic variants in *BAP1* on the risk of developing different types of cancer is largely unknown. This novel tumor suppressor is intriguing because of not only its known association with BRCA1 but also its established functions in the regulation of cell cycle, cell growth, and cell proliferation, among other key cellular functions. A recent report further established the potential role of *BAP1* in modulating the response to metabolic stress [34]. Prior mutational analyses of the *BAP1* gene used a family-based design and focused on the high risk individuals to identify rare variants with MAF <0.01. The sample sizes in these published mutational analyses of the *BAP1* gene were well below 100. In addition, various techniques such as comparative genomic hybridization array, targeted next-generation sequencing (NGS), whole exome NGS, sanger sequencing, and immunohistochemical analysis have been used to detect BAP1 mutations [35, 36]. However, due to the limitations of these techniques, detection of BAP1 mutation is still challenging and an ongoing research field. In this study, we performed an extensive investigation in different types of cancer that included nearly 10,000 cancer cases and 5,000 healthy controls to determine the association of the common germline genetic variants in *BAP1* with the risk of developing different types of cancer. TaqMan genotyping assay was used in this study which is quite mature and has been used in many small scale SNP genotyping studies and also in the validation of the top candidate SNPs from genome-wide association studies. The goal of this study is to establish the role of common variations as susceptibility loci for cancer, providing additional knowledge of the impact of this gene.

*BAP1* is located at chromosome 3p21.31-p21.2 in close vicinity to two other known genes, *DNAH1* (dynein, axonemal, heavy chain 1) and *PHF7* (PHD finger protein 7). This genomic region of interest, which was defined at 10 kilobases (kb) upstream and downstream of the BAP1 gene for a total of 28,985 base pairs (bp), contains a total of 135 common germline genetic variants that were genotyped as part of the HapMap project (Phase 3 data). Among white population, four tagging SNPs were identified, representing common genetic variation across this entire genomic region of interest. We also genotyped two other variants in this region identified in dbSNP owing to the potential functional roles of these variants. rs123598 is located in the 3’-UTR of *BAP1* and rs56238158 was identified as a non-synonymous variant in the whole genome sequencing of J. Craig Venter [37]. The rs56238158 variant was not polymorphic in our population of nearly 15,000 individuals, suggesting that it is a private variant or one occurring with a very low frequency in the population. The overall lack of genetic variation in this region is intriguing, particularly when considering the potential role of BAP1 in the tumorigenic process for a number of cancers.

In the risk analysis by cancer site, the most significant association was for rs11708581 in renal cell carcinoma, resulting in a 26% reduction in risk that remained significant after Bonferroni correction for multiple comparisons. We also found that rs390802 was associated with a decreased risk of renal cell carcinoma. These two SNPs are located within 10 kb downstream of *BAP1*. Because of the close proximity of other genes to *BAP1*, rs11708581 is a synonymous variant of *DNAH1* and rs390802 is an intronic variant of *DNAH1*. Using HaploReg [38] and RegulomeDB [39] from the ENCyclopedia Of DNA Elements (ENCODE) project data [40], we found that two regions covering these SNPs and their correlated variants (r^2^>0.8) had a length of 250 kb for rs11708581, including 80 SNPs and 224 kb for rs390802, including 56 SNPs (Supplementary Tables 2 and 3). Interestingly, rs123602 (an intronic variant of *BAP1*) and rs498946 (61 bps 3’ of BAP1) are in high linkage disequilibrium (LD) with rs390802 (r^2^=0.98 and 0.95, respectively) and moderate LD with rs11708581 (r^2^=0.56 and 0.56, respectively). Because genotyping data for rs11708581 and rs390802 were not available in TCGA for renal cell carcinoma, we used the proxy SNPs in the eQTL analysis and found that two proxy SNPs for rs11708581 and one proxy SNPs for rs390802 exhibited an significantly negative association with the expression level of BAP1. Genotyping data for rs123602 were available but did not show any association with BAP1 expression. Both rs11708581 and rs390802 are in the DNase I hypersensitivity region which is a signature of open chromatin that is typically associated with transcriptionally active regions of the genome [41]. Therefore, it is possible that rs11708581 and rs390802 affect the risk of developing renal cell carcinoma through *BAP1*.

We also found that rs12163565 was associated with an increased risk of developing lung cancer. Similar to rs11708581, rs12163565 is a missense variant of *DNAH1* and is located within 10kb of BAP. The region covering its correlated variants (r^2^>0.8) had a length of 201 kb, including five SNPs and none of the SNPs in the *BAP1* gene had high LD with rs12163565 (Supplementary Figure 1). Therefore, its role on the functional consequence of BAP1 remains unknown.

Overall, the results of this analysis of BAP1 common germline genetic variations in a large population of nearly 10,000 patients with cancer from five different cancer sites support the idea that common germline genetic variants in *BAP1* playing a role in mediating the risk of developing renal cell carcinoma and lung cancer.

## MATERIALS AND METHODS

### Study population

Cancer patients with newly diagnosed, histologically confirmed bladder cancer, esophageal cancer, renal cell carcinoma, lung cancer, or colorectal cancer were recruited from the University of Texas MD Anderson Cancer Center. Additional patients with colorectal cancer were recruited through the TexGen Consortium which focused on cancer patients seen at institutions throughout the Texas Medical Center. There were no recruitment restrictions on age, sex, ethnicity or cancer stage. Control subjects were defined as individuals with no prior history of cancer, excluding non-melanoma skin cancer, and were recruited in parallel with cases through two mechanisms: 1) random digit dialing and 2) from Kelsey-Seybold Clinics, the largest multi-site and multispecialty physician group in Houston, Texas. Details regarding the case and control recruitment for the on-going studies were described elsewhere [42–46]. Controls were frequency matched to cases by sex, age (±5), and ethnicity for all cancer types except for lung cancer, which was additionally matched by smoking status. All study participants provided written informed consent and the study was approved by the Institutional Review Boards of MD Anderson Cancer Center, Baylor College of Medicine, and Kelsey-Seybold Clinic. The analysis was restricted to self-reported white patients to minimize confounding by ethnicity.

### Data collection

A standardized questionnaire was used to collect epidemiologic information such as age, sex, ethnicity, medical history, and smoking history. A never smoker was defined as an individual who had smoked fewer than 100 cigarettes in his or her life, a former smoker was an individual who had smoked more than 100 cigarettes, but had quit smoking more than 1 year prior to diagnosis for cases and interview for controls. And a current smoker was an individual who was currently smoking or quit less than 1 year prior to diagnosis for cases or interview for controls, respectively. For the purposes of the current study, former and current smokers were grouped together as ever smokers. A blood specimen was collected from all study participants for genetic analyses. DNA was isolated from each blood sample using the QIAamp DNA Blood Maxi Kit (QIAGEN, Valencia, CA) following standard procedures and stored at -80 °C until use.

### SNP selection and genotyping

International HapMap Project data for the CEU population was used to select tagging SNPs within 10 kb of *BAP1* with an r^2^ of 0.80 and a minor allele frequency of >5%. Four variants were identified as tagging SNPs and an additional two variants were selected based on the basis of putative function: rs123598 and rs56238158 (Table 5). TaqMan genotyping assays for each SNP were purchased from ABI (Carlsbad, CA) and genotyping was completed following standard protocol. All genotyping was performed blinded to the status of each patient (i.e., case or control). Water negative controls and internal controls were included in each plate, and 5% of the samples were randomly selected and run in duplicate, with 100% concordance. The call rates were over 95% for all of the selected polymorphisms except for rs56238158, which was not detectable as a polymorphism in any of our study populations and was excluded from further analysis. The remaining five SNPs were tested for agreement with the Hardy–Weinberg using a goodness of-fit χ^2^ test in the control genotyping data.

**Table 5:** Genotyped genetic variants for *BAP1*

SNP	Chromosome (hg36)	Position	Nucleotide change	MAF*
rs11708581	chr3: 52428988	3’ Flanking	C>A	0.05
rs12163565	chr3: 52430526	3’ Flanking	G>A	0.17
rs390802	chr3: 52431671	3’ Flanking	G>A	0.13
rs123598	chr3: 52435860	3’ UTR	G>A	0.03
rs56238158	chr3: 52439302	Glu>Lys	C>T	0.13
rs13094687	chr3: 52450043	5’ Flanking	A>G	0.29

*MAF – minor allele frequency.

### Statistical analysis

We compared the difference in the distribution of categorical variables (sex and smoking status) using the Pearson’s χ^2^ test and the distribution of continuous variable (age) using the Student’s t-test. The association of each SNP with the risk of developing different types of cancer was estimated as ORs with 95% CIs using unconditional multivariate logistic regression, adjusting for age, sex, and smoking status for all five cancer types. The effect of rs11708581, rs123598, rs12163565, rs390802, and rs13094687 on the risk of developing different types of cancer was assessed using the dominant model. Stratified analyses were also performed to identify the effects of genetic variants in specific subgroups. The likelihood ratio test was used to assess the interaction of the genetic variants and the stratification variables by comparing the model with and the model without the interaction term. Gene track, H3K27AC mark, DNAse clusters, and transcription factor ChIP-seq plots in Supplementary Figure 1 were generated by UCSC genome browsers (http://genome.ucsc.edu) [47]. LD plot in Supplementary Figure 1 was generated by Haploview software [48] using European subjects from the 1000 Genome reference panel (phase I, April 2012). Statistical analyses were completed using the STATA software package (version 10, STATA, College Station, TX) and p-value<0.05 was considered statistically significant.

### Expression quantitative trait loci (eQTL) analysis

We performed eQTL analysis for SNPs rs11708581 and rs390802 to elucidate potential mechanism underlying the association of these SNPs with renal cell carcinoma. Data on renal clear cell carcinoma were downloaded from The Cancer Genome Atlas (TCGA, dbGaP Study Accession: phs000178.v9.p8, data portal: https://tcga-data.nci.nih.gov/tcga/tcgaHome2.jsp). Expression data (RNA-seq) and methylation (HumanMethylation450 chip) were measured in tumor samples, somatic copy number was inferred from the tumor samples and paired normal controls (Affymetrix SNP 6.0 platform), and genotyping data were measured from blood derived DNA samples (Affymetrix SNP 6.0 platform). For the *BAP1* gene, expression data were available for 533 cases, methylation data for the 19 CpG site were available for 319 cases, copy number data were available for 528 cases, and genotyping data were available for 556 cases. Expression values for BAP1 were log_2_-transformed. Because rs11708581 and rs390802 were not directly genotyped, we found five proxy SNPs for rs11708581 and three proxy SNPs for rs390802 (r^2^>0.8) using LD information from the 1000 Genome project. In the dbGaP data, SNP rs6809248 did not show high LD with the other proxy SNPs for rs11708581 (r^2^ range from 0.0 to 0.26) and was removed from further analysis. Following Li et al 2013 [49], We first evaluated the effect of somatic copy number and methylation level on log_2_-transformed expression level using linear regression. Univariate analysis identified the significant associations of somatic copy number and methylation levels of five CpG sites with BAP1 expression level. However, in the multivariate model including the somatic copy number and methylation levels of five CpG sites, only somatic copy number remained significant and none of the methylation levels were signficant. Therefore, we assessed the association of eight SNPs in the dominant model with the expression level of BAP1 using a linear regression model while adjusting for the effect of somatic copy number.

## SUPPLEMENTARY MATERIALS FIGURE AND TABLES





## References

[1] Carbone M, Yang H, Pass HI, Krausz T, Testa JR, Gaudino G (2013). BAP1 and cancer. Nat Rev Cancer.

[2] Jensen DE, Proctor M, Marquis ST, Gardner HP, Ha SI, Chodosh LA, Ishov AM, Tommerup N, Vissing H, Sekido Y, Minna J, Borodovsky A, Schultz DC (1998). BAP1: a novel ubiquitin hydrolase which binds to the BRCA1 RING finger and enhances BRCA1-mediated cell growth suppression. Oncogene.

[3] Mallery DL, Vandenberg CJ, Hiom K (2002). Activation of the E3 ligase function of the BRCA1/BARD1 complex by polyubiquitin chains. EMBO J.

[4] Ventii KH, Devi NS, Friedrich KL, Chernova TA, Tighiouart M, Van Meir EG, Wilkinson KD (2008). BRCA1-associated protein-1 is a tumor suppressor that requires deubiquitinating activity and nuclear localization. Cancer Res.

[5] Misaghi S, Ottosen S, Izrael-Tomasevic A, Arnott D, Lamkanfi M, Lee J, Liu J, O’Rourke K, Dixit VM, Wilson AC (2009). Association of C-terminal ubiquitin hydrolase BRCA1-associated protein 1 with cell cycle regulator host cell factor 1. Mol Cell Biol.

[6] Yu H, Mashtalir N, Daou S, Hammond-Martel I, Ross J, Sui G, Hart GW, Rauscher FJ, Drobetsky E, Milot E, Shi Y, Affar el B (2010). The ubiquitin carboxyl hydrolase BAP1 forms a ternary complex with YY1 and HCF-1 and is a critical regulator of gene expression. Mol Cell Biol.

[7] Bott M, Brevet M, Taylor BS, Shimizu S, Ito T, Wang L, Creaney J, Lake RA, Zakowski MF, Reva B, Sander C, Delsite R, Powell S (2011). The nuclear deubiquitinase BAP1 is commonly inactivated by somatic mutations and 3p21.1 losses in malignant pleural mesothelioma. Nat Genet.

[8] Yoshikawa Y, Sato A, Tsujimura T, Emi M, Morinaga T, Fukuoka K, Yamada S, Murakami A, Kondo N, Matsumoto S, Okumura Y, Tanaka F, Hasegawa S (2012). Frequent inactivation of the BAP1 gene in epithelioid-type malignant mesothelioma. Cancer Sci.

[9] Testa JR, Cheung M, Pei J, Below JE, Tan Y, Sementino E, Cox NJ, Dogan AU, Pass HI, Trusa S, Hesdorffer M, Nasu M, Powers A (2011). Germline BAP1 mutations predispose to malignant mesothelioma. Nat Genet.

[10] Harbour JW, Onken MD, Roberson ED, Duan S, Cao L, Worley LA, Council ML, Matatall KA, Helms C, Bowcock AM (2010). Frequent mutation of BAP1 in metastasizing uveal melanomas. Science.

[11] Abdel-Rahman MH, Pilarski R, Cebulla CM, Massengill JB, Christopher BN, Boru G, Hovland P, Davidorf FH (2011). Germline BAP1 mutation predisposes to uveal melanoma, lung adenocarcinoma, meningioma, and other cancers. J Med Genet.

[12] Njauw CN, Kim I, Piris A, Gabree M, Taylor M, Lane AM, DeAngelis MM, Gragoudas E, Duncan LM, Tsao H (2012). Germline BAP1 inactivation is preferentially associated with metastatic ocular melanoma and cutaneous-ocular melanoma families. PLoS One.

[13] Duns G, Hofstra RM, Sietzema JG, Hollema H, van Duivenbode I, Kuik A, Giezen C, Jan O, Bergsma JJ, Bijnen H, van der Vlies P, van den Berg E, Kok K (2012). Targeted exome sequencing in clear cell renal cell carcinoma tumors suggests aberrant chromatin regulation as a crucial step in ccRCC development. Hum Mutat.

[14] Pena-Llopis S, Vega-Rubin-de-Celis S, Liao A, Leng N, Pavia-Jimenez A, Wang S, Yamasaki T, Zhrebker L, Sivanand S, Spence P, Kinch L, Hambuch T, Jain S (2012). BAP1 loss defines a new class of renal cell carcinoma. Nat Genet.

[15] Wiesner T, Obenauf AC, Murali R, Fried I, Griewank KG, Ulz P, Windpassinger C, Wackernagel W, Loy S, Wolf I, Viale A, Lash AE, Pirun M (2011). Germline mutations in BAP1 predispose to melanocytic tumors. Nat Genet.

[16] Siegel R, Naishadham D, Jemal A (2013). Cancer statistics, 2013. CA Cancer J Clin.

[17] Amos CI, Wu X, Broderick P, Gorlov IP, Gu J, Eisen T, Dong Q, Zhang Q, Gu X, Vijayakrishnan J, Sullivan K, Matakidou A, Wang Y (2008). Genome-wide association scan of tag SNPs identifies a susceptibility locus for lung cancer at 15q25.1. Nat Genet.

[18] Thorgeirsson TE, Geller F, Sulem P, Rafnar T, Wiste A, Magnusson KP, Manolescu A, Thorleifsson G, Stefansson H, Ingason A, Stacey SN, Bergthorsson JT, Thorlacius S (2008). A variant associated with nicotine dependence, lung cancer and peripheral arterial disease. Nature.

[19] Hung RJ, McKay JD, Gaborieau V, Boffetta P, Hashibe M, Zaridze D, Mukeria A, Szeszenia-Dabrowska N, Lissowska J, Rudnai P, Fabianova E, Mates D, Bencko V (2008). A susceptibility locus for lung cancer maps to nicotinic acetylcholine receptor subunit genes on 15q25. Nature.

[20] Wang Y, Broderick P, Webb E, Wu X, Vijayakrishnan J, Matakidou A, Qureshi M, Dong Q, Gu X, Chen WV, Spitz MR, Eisen T, Amos CI (2008). Common 5p15.33 and 6p21.33 variants influence lung cancer risk. Nat Genet.

[21] Zanke BW, Greenwood CM, Rangrej J, Kustra R, Tenesa A, Farrington SM, Prendergast J, Olschwang S, Chiang T, Crowdy E, Ferretti V, Laflamme P, Sundararajan S (2007). Genome-wide association scan identifies a colorectal cancer susceptibility locus on chromosome 8q24. Nat Genet.

[22] Tomlinson I, Webb E, Carvajal-Carmona L, Broderick P, Kemp Z, Spain S, Penegar S, Chandler I, Gorman M, Wood W, Barclay E, Lubbe S, Martin L (2007). A genome-wide association scan of tag SNPs identifies a susceptibility variant for colorectal cancer at 8q24.21. Nat Genet.

[23] Broderick P, Carvajal-Carmona L, Pittman AM, Webb E, Howarth K, Rowan A, Lubbe S, Spain S, Sullivan K, Fielding S, Jaeger E, Vijayakrishnan J, Kemp Z (2007). A genome-wide association study shows that common alleles of SMAD7 influence colorectal cancer risk. Nat Genet.

[24] Tomlinson IP, Webb E, Carvajal-Carmona L, Broderick P, Howarth K, Pittman AM, Spain S, Lubbe S, Walther A, Sullivan K, Jaeger E, Fielding S, Rowan A (2008). A genome-wide association study identifies colorectal cancer susceptibility loci on chromosomes 10p14 and 8q23.3. Nat Genet.

[25] Tenesa A, Farrington SM, Prendergast JG, Porteous ME, Walker M, Haq N, Barnetson RA, Theodoratou E, Cetnarskyj R, Cartwright N, Semple C, Clark AJ, Reid FJ (2008). Genome-wide association scan identifies a colorectal cancer susceptibility locus on 11q23 and replicates risk loci at 8q24 and 18q21. Nat Genet.

[26] Wu X, Ye Y, Kiemeney LA, Sulem P, Rafnar T, Matullo G, Seminara D, Yoshida T, Saeki N, Andrew AS, Dinney CP, Czerniak B, Zhang ZF (2009). Genetic variation in the prostate stem cell antigen gene PSCA confers susceptibility to urinary bladder cancer. Nat Genet.

[27] Kiemeney LA, Thorlacius S, Sulem P, Geller F, Aben KK, Stacey SN, Gudmundsson J, Jakobsdottir M, Bergthorsson JT, Sigurdsson A, Blondal T, Witjes JA, Vermeulen SH (2008). Sequence variant on 8q24 confers susceptibility to urinary bladder cancer. Nat Genet.

[28] Rothman N, Garcia-Closas M, Chatterjee N, Malats N, Wu X, Figueroa JD, Real FX, Van Den Berg D, Matullo G, Baris D, Thun M, Kiemeney LA, Vineis P (2010). A multi-stage genome-wide association study of bladder cancer identifies multiple susceptibility loci. Nat Genet.

[29] Henrion M, Frampton M, Scelo G, Purdue M, Ye Y, Broderick P, Ritchie A, Kaplan R, Meade A, McKay J, Johansson M, Lathrop M, Larkin J (2013). Common variation at 2q22.3 (ZEB2) influences the risk of renal cancer. Hum Mol Genet.

[30] Wu X, Scelo G, Purdue MP, Rothman N, Johansson M, Ye Y, Wang Z, Zelenika D, Moore LE, Wood CG, Prokhortchouk E, Gaborieau V, Jacobs KB (2012). A genome-wide association study identifies a novel susceptibility locus for renal cell carcinoma on 12p11.23. Hum Mol Genet.

[31] Spencer C, Hechter E, Vukcevic D, Donnelly P (2011). Quantifying the underestimation of relative risks from genome-wide association studies. PLoS Genet.

[32] Ma XY, Zhang B, Zheng W (2014). Genetic variants associated with colorectal cancer risk: comprehensive research synopsis, meta-analysis, and epidemiological evidence. Gut.

[33] Chang CQ, Yesupriya A, Rowell JL, Pimentel CB, Clyne M, Gwinn M, Khoury MJ, Wulf A, Schully SD (2014). A systematic review of cancer GWAS and candidate gene meta-analyses reveals limited overlap but similar effect sizes. Eur J Hum Genet.

[34] Dai FY, Lee H, Zhang Y, Zhuang L, Yao H, Xi YX, Xiao ZD, You MJ, Li W, Su XP, Gan B (2017). BAP1 inhibits the ER stress gene regulatory network and modulates metabolic stress response. Proc Natl Acad Sci U S A.

[35] Nasu M, Emi M, Pastorino S, Tanji M, Powers A, Luk H, Baumann F, Zhang YA, Gazdar A, Kanodia S, Tiirikainen M, Flores E, Gaudino G (2015). High incidence of somatic BAP1 alterations in sporadic malignant mesothelioma. J Thorac Oncol.

[36] Yoshikawa Y, Emi M, Hashimoto-Tamaoki T, Ohmuraya M, Sato A, Tsujimura T, Hasegawa S, Nakano T, Nasu M, Pastorino S, Szymiczek A, Bononi A, Tanji M (2016). High-density array-CGH with targeted NGS unmask multiple noncontiguous minute deletions on chromosome 3p21 in mesothelioma. Proc Natl Acad Sci U S A.

[37] Levy S, Sutton G, Ng PC, Feuk L, Halpern AL, Walenz BP, Axelrod N, Huang J, Kirkness EF, Denisov G, Lin Y, MacDonald JR, Pang AW (2007). The diploid genome sequence of an individual human. PLoS Biol.

[38] Ward LD, Kellis M (2012). HaploReg: a resource for exploring chromatin states, conservation, and regulatory motif alterations within sets of genetically linked variants. Nucleic Acids Res.

[39] Boyle AP, Hong EL, Hariharan M, Cheng Y, Schaub MA, Kasowski M, Karczewski KJ, Park J, Hitz BC, Weng S, Cherry JM, Snyder M (2012). Annotation of functional variation in personal genomes using RegulomeDB. Genome Res.

[40] Dunham I, Kundaje A, Aldred SF, Collins PJ, Davis CA, Doyle F, Epstein CB, Frietze S, Harrow J, Kaul R, Khatun J, Lajoie BR, Landt SG (2012). An integrated encyclopedia of DNA elements in the human genome. Nature.

[41] Cockerill PN (2011). Structure and function of active chromatin and DNase I hypersensitive sites. FEBS J.

[42] Wu X, Lin J, Grossman HB, Huang M, Gu J, Etzel CJ, Amos CI, Dinney CP, Spitz MR (2007). Projecting individualized probabilities of developing bladder cancer in white individuals. J Clin Oncol.

[43] Pan J, Lin J, Izzo JG, Liu Y, Xing J, Huang M, Ajani JA, Wu X (2009). Genetic susceptibility to esophageal cancer: the role of the nucleotide excision repair pathway. Carcinogenesis.

[44] Clague J, Lin J, Cassidy A, Matin S, Tannir NM, Tamboli P, Wood CG, Wu X (2009). Family history and risk of renal cell carcinoma: results from a case-control study and systematic meta-analysis. Cancer Epidemiol Biomarkers Prev.

[45] Spitz MR, Hong WK, Amos CI, Wu X, Schabath MB, Dong Q, Shete S, Etzel CJ (2007). A risk model for prediction of lung cancer. J Natl Cancer Inst.

[46] Hildebrandt MA, Reyes ME, Lin MB, He YG, Nguyen SV, Hawk ET, Wu XF (2016). Germline genetic variants in the wnt/beta-catenin pathway as predictors of colorectal cancer risk. Cancer Epidemiol Biomarkers Prev.

[47] Kent WJ, Sugnet CW, Furey TS, Roskin KM, Pringle TH, Zahler AM, Haussler D (2002). The human genome browser at UCSC. Genome Res.

[48] Barrett JC, Fry B, Maller J, Daly MJ (2005). Haploview: analysis and visualization of LD and haplotype maps. Bioinformatics.

[49] Li QY, Seo JH, Stranger B, McKenna A, Pe’er I, LaFramboise T, Brown M, Tyekucheva S, Freedman ML (2013). Integrative eQTL-based analyses reveal the biology of breast cancer risk loci. Cell.

