# Supplementary material for: Common, germline genetic variations in the novel tumor suppressor BAP1 and risk of developing different types of cancer
Source: Oncotarget. 2017 Aug 24;8(43):74936–46. doi: 10.18632/oncotarget.20465 (PMC5650391; doi:10.18632/oncotarget.20465)
Supplement: Supplementary file 1 [file oncotarget-08-74936-s001.pdf]

Common, germline genetic variations in the novel tumor suppressor *BAP1* and risk of developing different types of cancer

SUPPLEMENTARY MATERIALS

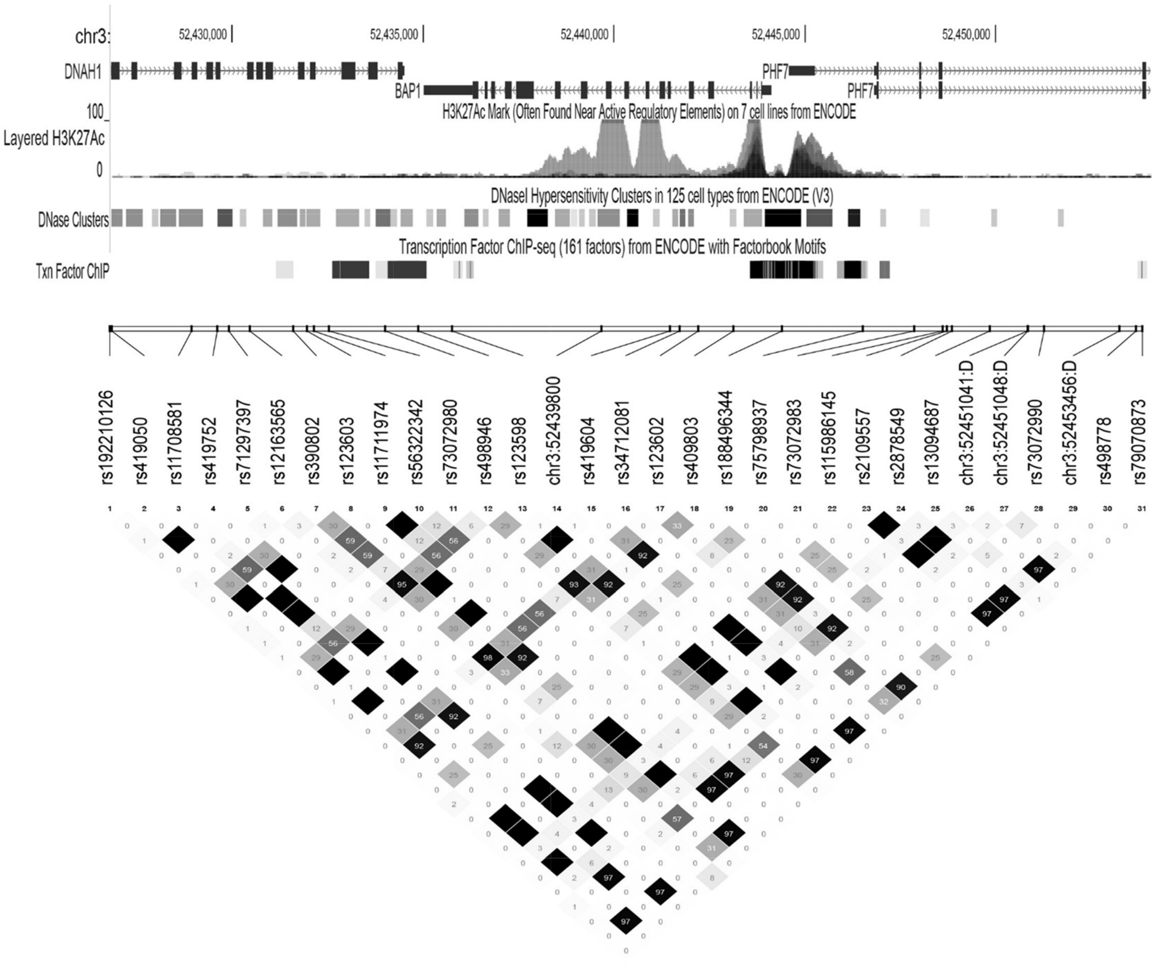

Supplementary Figure 1: Genome annotation and LD structure of the region covering BAP1.

Supplementary Table 1: Distribution of sex and smoking in each cancer site

Variables	Cases		P	Controls		P
	Male, n(%)	Female, n(%)		Male, n(%)	Female, n(%)	
**Bladder cancer**						
Never smoker	236(24.53)	104(40.78)		377(32.95)	114(39.58)	
Ever smoker	726(75.47)	151(59.22)	2.7E-07	767(67.05)	174(60.42)	0.03
**Colorectal cancer**						
Never smoker	647(47.93)	623(62.93)		506(37.59)	393(39.90)	
Ever smoker	703(52.07)	367(37.07)	6.1E-13	840(62.41)	592(60.10)	0.26
**Esophageal cancer**						
Never smoker	228(26.15)	42(36.21)		342(33.40)	56(41.79)	
Ever smoker	644(73.85)	74(63.79)	0.02	682(66.60)	78(58.21)	0.05
**Renal cell carcinoma**						
Never smoker	341(40.31)	239(56.77)		319(36.21)	169(38.32)	
Ever smoker	505(59.69)	182(43.23)	3E-08	562(63.79)	272(61.68)	0.45
**Lung cancer**						
Never smoker	205(11.24)	384(25.79)		205(11.24)	384(25.79)	
Ever smoker	1619(88.76)	1105(74.21)	1.2E-27	1619(88.76)	1105(74.21)	1.2E-27

Supplementary Table 2: HaploReg v2 analysis of rs11708581, rs390802, and rs12163565 and their correlated variants (r2>0.80)

See Supplementary File 1

Supplementary Table 3: Regulome analysis of rs11708581, rs390802, and rs12163565

#chromosome	coordinate	rsid	hits	score
chr3	52428987	rs11708581	Chromatin_Structure|DNase-seq	TF binding or DNase peak
chr3	52430525	rs12163565	Chromatin_Structure|DNase-seq	TF binding or DNase peak
chr3	52431670	rs390802	Chromatin_Structure|DNase-seq	TF binding or DNase peak
