# Supplementary material for: Common, germline genetic variations in the novel tumor suppressor BAP1 and risk of developing different types of cancer
Source: Oncotarget. 2017 Aug 24;8(43):74936–46. doi: 10.18632/oncotarget.20465 (PMC5650391; doi:10.18632/oncotarget.20465)
Supplement: Supplementary file 2 [file oncotarget-08-74936-s002.docx]

Supplementary Table 2. HaploReg v2 analysis of rs11708581, rs390802, and rs12163565 and their correlated variants (r2>0.80)

| **chr** | **pos (hg19)** | **LD** | **LD** | **variant** | **Ref** | **Alt** | **AFR** | **AMR** | **ASN** | **EUR** | **SiPhy** | **Promoter** | **Enhancer** | **DNAse** | **Proteins** | **eQTL** | **Motifs** | **GENCODE** | **dbSNP** |
| --- | --- | --- | --- | --- | --- | --- | --- | --- | --- | --- | --- | --- | --- | --- | --- | --- | --- | --- | --- |
|  |  | **(r²)** | **(D')** |  |  |  | **freq** | **freq** | **freq** | **freq** | **cons** | **histone marks** | **histone marks** |  | **bound** | **tissues** | **changed** | **genes** | **func annot** |
| **rs11708581 and variants with r^2^ >= 0.8** | | | | |  |  |  |  |  |  |  |  |  |  |  |  |  |  |  |
| 3 | 52269616 | 0.86 | 0.94 | [rs11715382](http://www.broadinstitute.org/mammals/haploreg/detail_v2.php?query=&id=rs11715382) | C | T | 0.15 | 0.09 | 0 | 0.1 |  |  | H1, GM12878 |  |  |  | 4 altered motifs | TWF2 | intronic |
| 3 | 52272028 | 0.9 | 0.96 | [rs7628461](http://www.broadinstitute.org/mammals/haploreg/detail_v2.php?query=&id=rs7628461) | A | C | 0.32 | 0.1 | 0 | 0.1 |  | GM12878, H1 | 7 cell types | 26 cell types | CTCF,ZBTB7A |  | AP-4,THAP1,VDR | TWF2 | intronic |
| 3 | 52274742 | 0.9 | 0.96 | [rs4082828](http://www.broadinstitute.org/mammals/haploreg/detail_v2.php?query=&id=rs4082828) | A | C | 0.33 | 0.1 | 0 | 0.1 |  | HepG2 | 8 cell types | 107 cell types | 11 bound proteins |  | DMRT4,E4BP4,Myc | RP5-1157M23.2 |  |
| 3 | 52276883 | 0.87 | 0.94 | [rs11719760](http://www.broadinstitute.org/mammals/haploreg/detail_v2.php?query=&id=rs11719760) | A | G | 0.27 | 0.11 | 0.01 | 0.1 |  |  |  |  |  |  | 5 altered motifs | 1.8kb 3' of RP5-1157M23.2 |  |
| 3 | 52276901 | 0.88 | 0.97 | [rs11711914](http://www.broadinstitute.org/mammals/haploreg/detail_v2.php?query=&id=rs11711914) | C | T | 0.25 | 0.1 | 0 | 0.09 |  |  |  |  |  |  | Ets,RXRA,SP1 | 1.8kb 3' of RP5-1157M23.2 |  |
| 3 | 52278241 | 0.91 | 0.97 | [rs7638204](http://www.broadinstitute.org/mammals/haploreg/detail_v2.php?query=&id=rs7638204) | C | T | 0.32 | 0.1 | 0 | 0.1 |  | H1 | 4 cell types | 21 cell types | 5 bound proteins |  | 19 altered motifs | 1.6kb 5' of PPM1M |  |
| 3 | 52279951 | 0.87 | 0.94 | [rs114645176](http://www.broadinstitute.org/mammals/haploreg/detail_v2.php?query=&id=rs114645176) | T | G | 0.3 | 0.11 | 0.01 | 0.1 |  | 7 cell types | Huvec, NHEK | 61 cell types | 7 bound proteins |  | 5 altered motifs | PPM1M | synonymous |
| 3 | 52280378 | 0.91 | 0.97 | [rs11715391](http://www.broadinstitute.org/mammals/haploreg/detail_v2.php?query=&id=rs11715391) | C | T | 0.31 | 0.1 | 0 | 0.1 |  | 7 cell types | Huvec, HMEC | 6 cell types | ERALPHA_A |  | 7 altered motifs | PPM1M | intronic |
| 3 | 52286349 | 0.87 | 0.94 | [rs11706541](http://www.broadinstitute.org/mammals/haploreg/detail_v2.php?query=&id=rs11706541) | G | C | 0.33 | 0.11 | 0.01 | 0.1 |  |  |  |  |  |  | 9 altered motifs | 1.7kb 3' of PPM1M |  |
| 3 | 52286585 | 0.91 | 0.97 | [rs73088773](http://www.broadinstitute.org/mammals/haploreg/detail_v2.php?query=&id=rs73088773) | G | A | 0.27 | 0.1 | 0 | 0.1 |  |  |  |  |  |  |  | 1.9kb 3' of WDR82 |  |
| 3 | 52286886 | 0.91 | 0.97 | [rs76103861](http://www.broadinstitute.org/mammals/haploreg/detail_v2.php?query=&id=rs76103861) | C | G | 0 | 0.08 | 0 | 0.1 |  |  |  |  |  |  | 8 altered motifs | 1.6kb 3' of WDR82 |  |
| 3 | 52290474 | 0.9 | 0.96 | [rs1767](http://www.broadinstitute.org/mammals/haploreg/detail_v2.php?query=&id=rs1767) | C | G | 0.33 | 0.11 | 0.01 | 0.1 |  |  |  |  |  |  | Mef2,RXRA,TATA | WDR82 | 3'-UTR |
| 3 | 52290550 | 0.94 | 0.98 | [rs1769](http://www.broadinstitute.org/mammals/haploreg/detail_v2.php?query=&id=rs1769) | A | G | 0.27 | 0.1 | 0 | 0.1 |  |  |  |  |  |  |  | WDR82 | 3'-UTR |
| 3 | 52293312 | 0.9 | 0.96 | [rs55847657](http://www.broadinstitute.org/mammals/haploreg/detail_v2.php?query=&id=rs55847657) | C | G | 0.33 | 0.11 | 0.01 | 0.1 |  |  | Huvec | 29 cell types | CFOS,P300 |  | GR,Nanog,Sox | WDR82 | intronic |
| 3 | 52293859 | 0.93 | 0.97 | [rs3210487](http://www.broadinstitute.org/mammals/haploreg/detail_v2.php?query=&id=rs3210487) | A | G | 0.27 | 0.1 | 0 | 0.1 |  |  |  |  |  |  |  | WDR82 | synonymous |
| 3 | 52294175 | 0.9 | 0.96 | [rs113895715](http://www.broadinstitute.org/mammals/haploreg/detail_v2.php?query=&id=rs113895715) | A | G | 0.34 | 0.11 | 0.01 | 0.1 |  |  |  |  |  |  |  | WDR82 | intronic |
| 3 | 52296022 | 0.9 | 0.96 | [rs140616356](http://www.broadinstitute.org/mammals/haploreg/detail_v2.php?query=&id=rs140616356) | TG | T | 0.33 | 0.11 | 0.01 | 0.1 |  |  |  |  |  |  | 7 altered motifs | WDR82 | intronic |
| 3 | 52298116 | 0.9 | 0.96 | [rs58531656](http://www.broadinstitute.org/mammals/haploreg/detail_v2.php?query=&id=rs58531656) | T | C | 0.33 | 0.11 | 0.01 | 0.1 |  |  |  |  |  |  | 4 altered motifs | WDR82 | intronic |
| 3 | 52298161 | 0.9 | 0.96 | [rs60382084](http://www.broadinstitute.org/mammals/haploreg/detail_v2.php?query=&id=rs60382084) | C | A | 0.33 | 0.11 | 0.01 | 0.1 |  |  |  |  |  |  | p300 | WDR82 | intronic |
| 3 | 52298986 | 0.9 | 0.96 | [rs6793317](http://www.broadinstitute.org/mammals/haploreg/detail_v2.php?query=&id=rs6793317) | T | C | 0.33 | 0.11 | 0.01 | 0.1 |  |  |  |  |  |  | Pou2f2,Pou3f2 | WDR82 | intronic |
| 3 | 52303213 | 0.94 | 0.98 | [rs7643913](http://www.broadinstitute.org/mammals/haploreg/detail_v2.php?query=&id=rs7643913) | G | A | 0.25 | 0.1 | 0 | 0.1 |  |  |  |  |  |  | 4 altered motifs | WDR82 | intronic |
| 3 | 52303600 | 0.94 | 0.98 | [rs11714358](http://www.broadinstitute.org/mammals/haploreg/detail_v2.php?query=&id=rs11714358) | G | C | 0.27 | 0.1 | 0 | 0.1 |  |  | Huvec |  |  |  | 5 altered motifs | WDR82 | intronic |
| 3 | 52305286 | 0.9 | 0.96 | [rs73088783](http://www.broadinstitute.org/mammals/haploreg/detail_v2.php?query=&id=rs73088783) | C | T | 0.33 | 0.11 | 0.01 | 0.1 |  | GM12878 | Huvec | HUVEC |  |  | Hmx,Isl2,Nkx3 | WDR82 | intronic |
| 3 | 52307291 | 0.94 | 0.98 | [rs144571993](http://www.broadinstitute.org/mammals/haploreg/detail_v2.php?query=&id=rs144571993) | A | T | 0.04 | 0.08 | 0 | 0.1 |  | GM12878 |  |  |  |  | 10 altered motifs | WDR82 | intronic |
| 3 | 52307496 | 0.94 | 0.98 | [rs61644644](http://www.broadinstitute.org/mammals/haploreg/detail_v2.php?query=&id=rs61644644) | T | C | 0.27 | 0.1 | 0 | 0.1 |  | GM12878 |  |  |  |  | TCF4 | WDR82 | intronic |
| 3 | 52308931 | 0.91 | 0.96 | [rs138592740](http://www.broadinstitute.org/mammals/haploreg/detail_v2.php?query=&id=rs138592740) | AAAAC | A | 0.39 | 0.11 | 0.01 | 0.1 |  |  | GM12878 |  |  |  | 4 altered motifs | WDR82 | intronic |
| 3 | 52309338 | 0.9 | 0.96 | [rs6809248](http://www.broadinstitute.org/mammals/haploreg/detail_v2.php?query=&id=rs6809248) | A | G | 0.33 | 0.11 | 0.01 | 0.1 |  | GM12878 |  | Hepatocytes |  |  | 7 altered motifs | WDR82 | intronic |
| 3 | 52309340 | 0.83 | 1 | [rs199878092](http://www.broadinstitute.org/mammals/haploreg/detail_v2.php?query=&id=rs199878092) | CAT | C | 0.04 | 0.07 | 0 | 0.08 |  | GM12878 |  | Hepatocytes |  |  | 10 altered motifs | WDR82 | intronic |
| 3 | 52309341 | 0.88 | 1 | [rs139103724](http://www.broadinstitute.org/mammals/haploreg/detail_v2.php?query=&id=rs139103724) | ATAT | A | 0.04 | 0.07 | 0 | 0.09 |  | GM12878 |  | Hepatocytes |  |  | Hoxa10,SRF,p300 | WDR82 | intronic |
| 3 | 52309827 | 0.94 | 0.98 | [rs11715208](http://www.broadinstitute.org/mammals/haploreg/detail_v2.php?query=&id=rs11715208) | G | A | 0.04 | 0.08 | 0 | 0.1 |  | 5 cell types | Huvec |  |  |  | 6 altered motifs | WDR82 | intronic |
| 3 | 52312976 | 0.94 | 0.98 | [rs77020200](http://www.broadinstitute.org/mammals/haploreg/detail_v2.php?query=&id=rs77020200) | C | T | 0.26 | 0.1 | 0 | 0.1 |  | 9 cell types |  | 7 cell types | 12 bound proteins |  | CTCF,RREB-1 | WDR82 |  |
| 3 | 52313214 | 0.9 | 0.96 | [rs73088785](http://www.broadinstitute.org/mammals/haploreg/detail_v2.php?query=&id=rs73088785) | T | G | 0.34 | 0.11 | 0.01 | 0.1 |  | 4 cell types | 5 cell types | Th2 |  |  | AFP1,Myc | WDR82 |  |
| 3 | 52313454 | 0.9 | 0.96 | [rs11718729](http://www.broadinstitute.org/mammals/haploreg/detail_v2.php?query=&id=rs11718729) | C | A | 0.34 | 0.11 | 0.01 | 0.1 |  | H1, HepG2, NHLF | 6 cell types | Adult_CD4_Th0,NHDF-Ad |  |  | Sin3Ak-20,Zbtb3 | WDR82 |  |
| 3 | 52318056 | 0.94 | 0.98 | [rs140771279](http://www.broadinstitute.org/mammals/haploreg/detail_v2.php?query=&id=rs140771279) | G | A | 0.27 | 0.1 | 0 | 0.1 |  |  | HepG2 |  |  |  | NF-kappaB,ZBTB33,ZEB1 | WDR82 |  |
| 3 | 52318281 | 0.94 | 0.98 | [rs73088791](http://www.broadinstitute.org/mammals/haploreg/detail_v2.php?query=&id=rs73088791) | C | A | 0.27 | 0.1 | 0 | 0.1 |  |  | HepG2 |  |  |  | BCL,Myc,NF-E2 | WDR82 |  |
| 3 | 52321688 | 0.94 | 0.98 | [rs149148515](http://www.broadinstitute.org/mammals/haploreg/detail_v2.php?query=&id=rs149148515) | GA | G | 0.26 | 0.1 | 0 | 0.1 |  | 9 cell types |  | Gliobla,HPDE6-E6E7,Medullo | 12 bound proteins |  | 7 altered motifs | GLYCTK |  |
| 3 | 52321976 | 0.94 | 0.98 | [rs11707056](http://www.broadinstitute.org/mammals/haploreg/detail_v2.php?query=&id=rs11707056) | C | A | 0.05 | 0.08 | 0 | 0.1 |  | 9 cell types |  | 16 cell types | 24 bound proteins |  | TLX1::NFIC,ZBRK1 | GLYCTK | intronic |
| 3 | 52323344 | 0.94 | 0.98 | [rs6783652](http://www.broadinstitute.org/mammals/haploreg/detail_v2.php?query=&id=rs6783652) | G | A | 0.27 | 0.1 | 0 | 0.1 |  | GM12878 | 7 cell types |  | GATA1,POL2 |  | 9 altered motifs | GLYCTK | intronic |
| 3 | 52325039 | 0.94 | 0.98 | [rs35976326](http://www.broadinstitute.org/mammals/haploreg/detail_v2.php?query=&id=rs35976326) | G | A | 0.34 | 0.1 | 0 | 0.1 |  | HepG2 |  |  |  |  | Ik-1 | GLYCTK | synonymous |
| 3 | 52328024 | 0.94 | 0.98 | [rs55676974](http://www.broadinstitute.org/mammals/haploreg/detail_v2.php?query=&id=rs55676974) | G | A | 0.34 | 0.1 | 0 | 0.1 |  |  |  |  |  |  | AP-2rep,Glis2 | GLYCTK | 3'-UTR |
| 3 | 52328533 | 0.9 | 0.96 | [rs3733063](http://www.broadinstitute.org/mammals/haploreg/detail_v2.php?query=&id=rs3733063) | G | A | 0.34 | 0.11 | 0.01 | 0.1 |  |  |  |  |  |  | 4 altered motifs | GLYCTK | 3'-UTR |
| 3 | 52328607 | 0.94 | 0.98 | [rs11708811](http://www.broadinstitute.org/mammals/haploreg/detail_v2.php?query=&id=rs11708811) | C | T | 0.11 | 0.08 | 0 | 0.1 |  |  | H1 |  |  |  | EWSR1-FLI1 | GLYCTK | 3'-UTR |
| 3 | 52331266 | 0.9 | 0.96 | [rs17052051](http://www.broadinstitute.org/mammals/haploreg/detail_v2.php?query=&id=rs17052051) | C | G | 0.34 | 0.11 | 0.01 | 0.1 |  |  | HSMM | H1-hESC | POL2 |  | TFE | GLYCTK-AS1 |  |
| 3 | 52331793 | 0.93 | 0.97 | [rs17052052](http://www.broadinstitute.org/mammals/haploreg/detail_v2.php?query=&id=rs17052052) | A | G | 0.34 | 0.1 | 0 | 0.1 |  | H1 | HepG2, K562 | GM19238 |  |  | Pou2f2,Zfp410 | GLYCTK-AS1 |  |
| 3 | 52332292 | 0.9 | 0.96 | [rs73088800](http://www.broadinstitute.org/mammals/haploreg/detail_v2.php?query=&id=rs73088800) | A | G | 0.33 | 0.11 | 0.01 | 0.1 |  | H1 | K562, HepG2 | 5 cell types |  |  | AhR::Arnt,Arnt,Myc | GLYCTK-AS1 |  |
| 3 | 52334593 | 0.9 | 0.96 | [rs17052053](http://www.broadinstitute.org/mammals/haploreg/detail_v2.php?query=&id=rs17052053) | T | C | 0.33 | 0.11 | 0.01 | 0.1 |  |  | 5 cell types | 4 cell types | POL2,GATA1 |  | Cdx2,ELF1,Irf | 1.5kb 5' of GLYCTK-AS1 |  |
| 3 | 52337754 | 0.94 | 0.98 | [rs17052054](http://www.broadinstitute.org/mammals/haploreg/detail_v2.php?query=&id=rs17052054) | C | T | 0.33 | 0.1 | 0 | 0.1 |  |  | K562 |  |  |  | LXR,VDR | 4.7kb 5' of GLYCTK-AS1 |  |
| 3 | 52338590 | 0.9 | 0.96 | [rs56352611](http://www.broadinstitute.org/mammals/haploreg/detail_v2.php?query=&id=rs56352611) | T | C | 0.33 | 0.11 | 0.01 | 0.1 |  |  |  | Osteobl |  |  | 4 altered motifs | 5.5kb 5' of GLYCTK-AS1 |  |
| 3 | 52341126 | 0.97 | 1 | [rs10461027](http://www.broadinstitute.org/mammals/haploreg/detail_v2.php?query=&id=rs10461027) | G | A | 0.18 | 0.09 | 0 | 0.1 |  |  | HepG2 | Melano |  |  | TATA | 8kb 5' of GLYCTK-AS1 |  |
| 3 | 52343679 | 0.97 | 1 | [rs10460962](http://www.broadinstitute.org/mammals/haploreg/detail_v2.php?query=&id=rs10460962) | C | T | 0.33 | 0.1 | 0 | 0.1 |  |  | GM12878 |  |  |  | 7 altered motifs | 6.7kb 5' of DNAH1 |  |
| 3 | 52343742 | 0.91 | 0.97 | [rs10461028](http://www.broadinstitute.org/mammals/haploreg/detail_v2.php?query=&id=rs10461028) | A | G | 0.34 | 0.11 | 0.02 | 0.1 |  |  | GM12878 |  |  |  | Maf,SREBP,Zbtb3 | 6.6kb 5' of DNAH1 |  |
| 3 | 52345459 | 0.96 | 0.99 | [rs6445385](http://www.broadinstitute.org/mammals/haploreg/detail_v2.php?query=&id=rs6445385) | A | G | 0.31 | 0.1 | 0 | 0.1 |  |  | GM12878 | 5 cell types |  |  |  | 4.9kb 5' of DNAH1 |  |
| 3 | 52351553 | 0.97 | 1 | [rs7615405](http://www.broadinstitute.org/mammals/haploreg/detail_v2.php?query=&id=rs7615405) | T | G | 0.31 | 0.1 | 0 | 0.1 |  | 5 cell types | HSMM | NHDF-Ad |  |  | 14 altered motifs | DNAH1 | intronic |
| 3 | 52354061 | 0.97 | 1 | [rs11717028](http://www.broadinstitute.org/mammals/haploreg/detail_v2.php?query=&id=rs11717028) | A | G | 0.18 | 0.09 | 0 | 0.1 |  |  | NHEK, HMEC | 4 cell types |  |  | Fox,Foxj1 | DNAH1 | intronic |
| 3 | 52359972 | 0.97 | 1 | [rs11710014](http://www.broadinstitute.org/mammals/haploreg/detail_v2.php?query=&id=rs11710014) | C | T | 0.31 | 0.1 | 0 | 0.1 |  |  |  | pHTE |  |  | AP-2 | DNAH1 | intronic |
| 3 | 52367172 | 1 | 1 | [rs55813842](http://www.broadinstitute.org/mammals/haploreg/detail_v2.php?query=&id=rs55813842) | G | C | 0.18 | 0.09 | 0 | 0.1 |  |  |  |  |  |  | Crx,Pitx2 | DNAH1 | intronic |
| 3 | 52379110 | 1 | 1 | [rs11713914](http://www.broadinstitute.org/mammals/haploreg/detail_v2.php?query=&id=rs11713914) | G | A | 0.05 | 0.08 | 0 | 0.1 |  |  |  | NHEK |  |  | Ets | DNAH1 | intronic |
| 3 | 52382822 | 1 | 1 | [rs7633687](http://www.broadinstitute.org/mammals/haploreg/detail_v2.php?query=&id=rs7633687) | C | T | 0.11 | 0.08 | 0 | 0.1 |  |  |  |  |  |  |  | DNAH1 | intronic |
| 3 | 52391473 | 1 | 1 | [rs57849650](http://www.broadinstitute.org/mammals/haploreg/detail_v2.php?query=&id=rs57849650) | G | A | 0.18 | 0.09 | 0 | 0.1 |  |  |  |  |  |  |  | DNAH1 | intronic |
| 3 | 52396944 | 1 | 1 | [rs11706515](http://www.broadinstitute.org/mammals/haploreg/detail_v2.php?query=&id=rs11706515) | G | A | 0.05 | 0.08 | 0 | 0.1 |  |  |  |  |  |  | EWSR1-FLI1,LBP-9,Rad21 | DNAH1 | intronic |
| 3 | 52397419 | 1 | 1 | [rs56899118](http://www.broadinstitute.org/mammals/haploreg/detail_v2.php?query=&id=rs56899118) | G | A | 0.27 | 0.09 | 0 | 0.1 |  |  |  | LNCaP,Hepatocytes,Osteobl |  |  | Myf,TAL1 | DNAH1 | intronic |
| 3 | 52397539 | 1 | 1 | [rs73091166](http://www.broadinstitute.org/mammals/haploreg/detail_v2.php?query=&id=rs73091166) | C | T | 0.01 | 0.08 | 0 | 0.1 |  |  |  | Hepatocytes |  |  | ERalpha-a | DNAH1 | intronic |
| 3 | 52397701 | 1 | 1 | [rs73091168](http://www.broadinstitute.org/mammals/haploreg/detail_v2.php?query=&id=rs73091168) | G | A | 0.27 | 0.09 | 0 | 0.1 |  |  |  | Fibrobl |  |  | Nr2f2,Pax-6,SP1 | DNAH1 | intronic |
| 3 | 52404111 | 1 | 1 | [rs746239](http://www.broadinstitute.org/mammals/haploreg/detail_v2.php?query=&id=rs746239) | G | A | 0.05 | 0.08 | 0 | 0.1 |  |  |  |  |  |  |  | DNAH1 | intronic |
| 3 | 52409421 | 1 | 1 | [rs56002041](http://www.broadinstitute.org/mammals/haploreg/detail_v2.php?query=&id=rs56002041) | A | G | 0 | 0.08 | 0 | 0.1 |  |  | GM12878 | HSMMtube,MCF-7 |  |  | 6 altered motifs | DNAH1 | missense |
| 3 | 52414639 | 1 | 1 | [rs55806905](http://www.broadinstitute.org/mammals/haploreg/detail_v2.php?query=&id=rs55806905) | C | G | 0.11 | 0.08 | 0 | 0.1 |  |  | K562 |  |  |  |  | DNAH1 | intronic |
| 3 | 52420414 | 1 | 1 | [rs11720365](http://www.broadinstitute.org/mammals/haploreg/detail_v2.php?query=&id=rs11720365) | G | A | 0.12 | 0.08 | 0 | 0.1 |  |  |  |  |  |  | TBX5 | DNAH1 | intronic |
| 3 | 52420901 | 1 | 1 | [rs11721277](http://www.broadinstitute.org/mammals/haploreg/detail_v2.php?query=&id=rs11721277) | G | A | 0.27 | 0.09 | 0 | 0.1 |  |  |  | Osteobl |  |  | Nkx2 | DNAH1 | intronic |
| 3 | 52428988 | 1 | 1 | [***rs11708581***](http://www.broadinstitute.org/mammals/haploreg/detail_v2.php?query=&id=rs11708581) | C | A | 0 | 0.08 | 0 | 0.1 |  |  |  | Th1,Medullo,Urothelia |  |  | 4 altered motifs | DNAH1 | synonymous |
| 3 | 52432214 | 1 | 1 | [rs11711974](http://www.broadinstitute.org/mammals/haploreg/detail_v2.php?query=&id=rs11711974) | G | A | 0.27 | 0.09 | 0 | 0.1 |  |  | HepG2 | Gliobla,Hepatocytes,PANC-1 |  |  | ELF1 | DNAH1 | intronic |
| 3 | 52432610 | 1 | 1 | [rs56322342](http://www.broadinstitute.org/mammals/haploreg/detail_v2.php?query=&id=rs56322342) | G | A | 0.27 | 0.09 | 0 | 0.1 |  |  |  |  |  |  | YY1 | DNAH1 | intronic |
| 3 | 52451474 | 0.94 | 0.98 | [rs73072990](http://www.broadinstitute.org/mammals/haploreg/detail_v2.php?query=&id=rs73072990) | T | C | 0.12 | 0.08 | 0 | 0.1 |  |  |  |  |  |  | Evi-1,Foxp1,Mef2 | PHF7 | intronic |
| 3 | 52454262 | 0.94 | 0.98 | [rs3886875](http://www.broadinstitute.org/mammals/haploreg/detail_v2.php?query=&id=rs3886875) | T | G | 0 | 0.08 | 0 | 0.1 |  |  |  |  |  |  |  | PHF7 | intronic |
| 3 | 52477866 | 0.83 | 0.94 | [rs11716487](http://www.broadinstitute.org/mammals/haploreg/detail_v2.php?query=&id=rs11716487) | T | C | 0.67 | 0.12 | 0 | 0.1 |  | HepG2 | Huvec, GM12878 | Osteobl,NB4 |  |  | 4 altered motifs | SEMA3G | intronic |
| 3 | 52478389 | 0.88 | 0.98 | [rs729555](http://www.broadinstitute.org/mammals/haploreg/detail_v2.php?query=&id=rs729555) | C | T | 0.12 | 0.08 | 0 | 0.09 |  | HepG2 | H1, Huvec | H1-hESC,LNCaP |  |  | 5 altered motifs | SEMA3G | intronic |
| 3 | 52481406 | 0.88 | 0.95 | [rs10865971](http://www.broadinstitute.org/mammals/haploreg/detail_v2.php?query=&id=rs10865971) | A | G | 0.14 | 0.08 | 0 | 0.1 |  |  | HepG2 |  | CTCF |  | 5 altered motifs | 2.3kb 5' of SEMA3G |  |
| 3 | 52488517 | 0.88 | 0.97 | [rs724801](http://www.broadinstitute.org/mammals/haploreg/detail_v2.php?query=&id=rs724801) | C | T | 0.04 | 0.08 | 0 | 0.09 |  | HepG2, HSMM | 4 cell types | 4 cell types |  |  | Evi-1,Hmbox1 | 430bp 5' of TNNC1 |  |
| 3 | 52490511 | 0.84 | 0.97 | [rs10658765](http://www.broadinstitute.org/mammals/haploreg/detail_v2.php?query=&id=rs10658765) | T | TGCCA | 0.14 | 0.08 | 0 | 0.09 |  | 9 cell types |  | 24 cell types | ZNF263 |  | 4 altered motifs | NISCH | intronic |
| 3 | 52494588 | 0.87 | 0.97 | [rs6776759](http://www.broadinstitute.org/mammals/haploreg/detail_v2.php?query=&id=rs6776759) | T | A | 0.28 | 0.09 | 0 | 0.09 |  |  |  |  |  |  | 6 altered motifs | NISCH | intronic |
| 3 | 52519854 | 0.87 | 0.97 | [rs17263770](http://www.broadinstitute.org/mammals/haploreg/detail_v2.php?query=&id=rs17263770) | G | C | 0.13 | 0.08 | 0 | 0.09 |  |  |  |  |  |  | Hoxa5 | NISCH | intronic |
| **rs390802 and variants with r^2^ >= 0.8** | | | | |  |  |  |  |  |  |  |  |  |  |  |  |  |  |  |
| 3 | 52299521 | 0.93 | 0.97 | [rs59612757](http://www.broadinstitute.org/mammals/haploreg/detail_v2.php?query=&id=rs59612757) | T | C | 0.42 | 0.18 | 0.03 | 0.16 |  |  |  | 9 cell types |  |  | CTCF | WDR82 | intronic |
| 3 | 52315192 | 0.92 | 0.96 | [rs9844183](http://www.broadinstitute.org/mammals/haploreg/detail_v2.php?query=&id=rs9844183) | A | G | 0.42 | 0.18 | 0.03 | 0.16 |  |  | HSMM |  |  |  | Pou1f1,Pou5f1,XBP-1 | WDR82 |  |
| 3 | 52324169 | 0.86 | 0.97 | [rs3796343](http://www.broadinstitute.org/mammals/haploreg/detail_v2.php?query=&id=rs3796343) | A | G | 0.46 | 0.17 | 0.03 | 0.15 |  | HepG2 | Huvec | CMK | 10 bound proteins |  | 5 altered motifs | GLYCTK | intronic |
| 3 | 52344680 | 0.95 | 0.98 | [rs17052058](http://www.broadinstitute.org/mammals/haploreg/detail_v2.php?query=&id=rs17052058) | A | G | 0.34 | 0.18 | 0.03 | 0.16 |  |  | GM12878 |  |  |  | Irf | 5.7kb 5' of DNAH1 |  |
| 3 | 52346240 | 0.95 | 0.98 | [rs17052061](http://www.broadinstitute.org/mammals/haploreg/detail_v2.php?query=&id=rs17052061) | T | G | 0.34 | 0.17 | 0.02 | 0.16 |  |  | GM12878 | Chorion |  |  | COMP1,E2F,GATA | 4.1kb 5' of DNAH1 |  |
| 3 | 52346730 | 0.92 | 0.98 | [rs111593386](http://www.broadinstitute.org/mammals/haploreg/detail_v2.php?query=&id=rs111593386) | G | T | 0.34 | 0.17 | 0.01 | 0.15 |  |  | GM12878 |  |  |  | Zbtb3 | 3.6kb 5' of DNAH1 |  |
| 3 | 52350212 | 0.96 | 0.99 | [rs6445386](http://www.broadinstitute.org/mammals/haploreg/detail_v2.php?query=&id=rs6445386) | A | G | 0.34 | 0.17 | 0.02 | 0.15 |  |  | GM12878, HSMM | 8 cell types |  |  | Zfp410 | 122bp 5' of DNAH1 |  |
| 3 | 52350745 | 0.96 | 0.99 | [rs55997317](http://www.broadinstitute.org/mammals/haploreg/detail_v2.php?query=&id=rs55997317) | G | A | 0.34 | 0.17 | 0.02 | 0.15 |  |  | GM12878 | Fibrobl,Myometr |  |  | BCL,HNF4 | DNAH1 | intronic |
| 3 | 52352864 | 0.93 | 0.99 | [rs17052068](http://www.broadinstitute.org/mammals/haploreg/detail_v2.php?query=&id=rs17052068) | C | T | 0.34 | 0.17 | 0.01 | 0.15 |  |  |  | ProgFib |  |  | 4 altered motifs | DNAH1 | intronic |
| 3 | 52354242 | 0.96 | 0.99 | [rs57441156](http://www.broadinstitute.org/mammals/haploreg/detail_v2.php?query=&id=rs57441156) | C | G | 0.33 | 0.17 | 0.02 | 0.15 |  |  | HMEC, NHEK | SK-N-SH_RA |  |  | AP-2,EBF,Roaz | DNAH1 | intronic |
| 3 | 52354376 | 0.93 | 0.99 | [rs61150019](http://www.broadinstitute.org/mammals/haploreg/detail_v2.php?query=&id=rs61150019) | G | A | 0.34 | 0.17 | 0.01 | 0.15 |  |  | NHEK | Caco-2,PrEC,SK-N-SH_RA |  |  | CTCF,Rad21 | DNAH1 | intronic |
| 3 | 52354432 | 0.96 | 0.99 | [rs55965068](http://www.broadinstitute.org/mammals/haploreg/detail_v2.php?query=&id=rs55965068) | A | G | 0.34 | 0.17 | 0.02 | 0.15 |  |  | NHEK | Caco-2 |  |  | SREBP | DNAH1 | intronic |
| 3 | 52354772 | 0.93 | 0.99 | [rs6763882](http://www.broadinstitute.org/mammals/haploreg/detail_v2.php?query=&id=rs6763882) | C | T | 0.33 | 0.17 | 0.01 | 0.15 |  |  |  | Osteobl |  |  | 8 altered motifs | DNAH1 | intronic |
| 3 | 52358117 | 0.93 | 0.97 | [rs11711934](http://www.broadinstitute.org/mammals/haploreg/detail_v2.php?query=&id=rs11711934) | T | C | 0.34 | 0.17 | 0.02 | 0.16 |  | HepG2 |  | 7 cell types | RXRA |  | ERalpha-a,GCNF,RORalpha1 | DNAH1 | intronic |
| 3 | 52358417 | 0.93 | 0.99 | [rs11707931](http://www.broadinstitute.org/mammals/haploreg/detail_v2.php?query=&id=rs11707931) | C | G | 0.34 | 0.17 | 0.01 | 0.15 |  | HepG2 |  | HSMM,HepG2,Gliobla |  |  | BCL,NF-kappaB | DNAH1 | intronic |
| 3 | 52359040 | 0.93 | 0.99 | [rs35133416](http://www.broadinstitute.org/mammals/haploreg/detail_v2.php?query=&id=rs35133416) | C | T | 0.34 | 0.17 | 0.01 | 0.15 |  |  |  | HSMMtube,GM12892,iPS |  |  | 9 altered motifs | DNAH1 | intronic |
| 3 | 52359678 | 0.94 | 0.97 | [rs6796333](http://www.broadinstitute.org/mammals/haploreg/detail_v2.php?query=&id=rs6796333) | T | C | 0.34 | 0.17 | 0.02 | 0.16 |  |  |  |  |  |  | PPAR | DNAH1 | intronic |
| 3 | 52366044 | 0.95 | 0.99 | [rs34762015](http://www.broadinstitute.org/mammals/haploreg/detail_v2.php?query=&id=rs34762015) | G | A | 0.34 | 0.17 | 0.01 | 0.15 |  |  |  |  |  |  | 9 altered motifs | DNAH1 | intronic |
| 3 | 52368387 | 0.95 | 0.99 | [rs2335640](http://www.broadinstitute.org/mammals/haploreg/detail_v2.php?query=&id=rs2335640) | T | C | 0.34 | 0.17 | 0.01 | 0.15 |  |  |  |  |  |  | Egr-1,Mrg1::Hoxa9,NF-E2 | DNAH1 | intronic |
| 3 | 52368947 | 0.98 | 0.99 | [rs34238085](http://www.broadinstitute.org/mammals/haploreg/detail_v2.php?query=&id=rs34238085) | A | C | 0.34 | 0.17 | 0.02 | 0.16 |  |  |  |  |  |  | 16 altered motifs | DNAH1 | intronic |
| 3 | 52371789 | 0.98 | 0.99 | [rs13059674](http://www.broadinstitute.org/mammals/haploreg/detail_v2.php?query=&id=rs13059674) | T | G | 0.34 | 0.17 | 0.02 | 0.16 |  |  | HSMM, NHLF |  |  |  | NRSF | DNAH1 | intronic |
| 3 | 52371991 | 0.94 | 0.99 | [rs11705963](http://www.broadinstitute.org/mammals/haploreg/detail_v2.php?query=&id=rs11705963) | T | G | 0.67 | 0.19 | 0.02 | 0.16 |  |  | NHLF, HSMM |  |  |  | Foxp1,HDAC2 | DNAH1 | intronic |
| 3 | 52372128 | 0.93 | 0.98 | [rs11706001](http://www.broadinstitute.org/mammals/haploreg/detail_v2.php?query=&id=rs11706001) | T | A | 0.31 | 0.17 | 0.01 | 0.15 |  |  | NHLF, HSMM |  |  |  | 44 altered motifs | DNAH1 | intronic |
| 3 | 52372366 | 0.94 | 0.99 | [rs11706108](http://www.broadinstitute.org/mammals/haploreg/detail_v2.php?query=&id=rs11706108) | T | C | 0.67 | 0.19 | 0.03 | 0.16 |  |  |  | 15 cell types |  |  | 5 altered motifs | DNAH1 | intronic |
| 3 | 52373792 | 0.93 | 0.98 | [rs7643570](http://www.broadinstitute.org/mammals/haploreg/detail_v2.php?query=&id=rs7643570) | G | T | 0.67 | 0.19 | 0.03 | 0.16 |  |  |  | 102 cell types | 11 bound proteins |  | 18 altered motifs | DNAH1 | intronic |
| 3 | 52374516 | 0.93 | 0.98 | [rs56357549](http://www.broadinstitute.org/mammals/haploreg/detail_v2.php?query=&id=rs56357549) | T | G | 0.34 | 0.16 | 0.01 | 0.15 |  |  |  | HL-60 |  |  | SREBP | DNAH1 | intronic |
| 3 | 52374889 | 0.9 | 0.95 | [rs6794675](http://www.broadinstitute.org/mammals/haploreg/detail_v2.php?query=&id=rs6794675) | T | A | 0.67 | 0.18 | 0.01 | 0.16 |  |  |  |  |  |  | 8 altered motifs | DNAH1 | intronic |
| 3 | 52375711 | 0.98 | 0.99 | [rs34523877](http://www.broadinstitute.org/mammals/haploreg/detail_v2.php?query=&id=rs34523877) | T | C | 0.34 | 0.17 | 0.02 | 0.16 |  |  |  | NHDF-Ad |  |  | 4 altered motifs | DNAH1 | intronic |
| 3 | 52377294 | 0.93 | 0.98 | [rs13091545](http://www.broadinstitute.org/mammals/haploreg/detail_v2.php?query=&id=rs13091545) | A | G | 0.67 | 0.19 | 0.02 | 0.16 |  |  |  |  |  |  |  | DNAH1 | intronic |
| 3 | 52379420 | 0.97 | 0.99 | [rs11718125](http://www.broadinstitute.org/mammals/haploreg/detail_v2.php?query=&id=rs11718125) | T | C | 0.33 | 0.16 | 0.02 | 0.16 |  |  |  |  |  |  | Bach1,Bach2,Pax-2 | DNAH1 | intronic |
| 3 | 52380991 | 0.94 | 0.99 | [rs11716017](http://www.broadinstitute.org/mammals/haploreg/detail_v2.php?query=&id=rs11716017) | C | T | 0.3 | 0.15 | 0.01 | 0.15 |  |  | 4 cell types | 44 cell types |  |  | 4 altered motifs | DNAH1 | intronic |
| 3 | 52383120 | 0.94 | 0.99 | [rs34355561](http://www.broadinstitute.org/mammals/haploreg/detail_v2.php?query=&id=rs34355561) | G | A | 0.29 | 0.15 | 0.01 | 0.15 |  |  |  | LNCaP |  |  | HIF1::Arnt | DNAH1 | intronic |
| 3 | 52385115 | 0.95 | 0.99 | [rs34908451](http://www.broadinstitute.org/mammals/haploreg/detail_v2.php?query=&id=rs34908451) | C | T | 0.3 | 0.16 | 0.01 | 0.15 |  |  |  |  |  |  | 12 altered motifs | DNAH1 | intronic |
| 3 | 52387078 | 0.94 | 0.99 | [rs35860312](http://www.broadinstitute.org/mammals/haploreg/detail_v2.php?query=&id=rs35860312) | T | C | 0.67 | 0.19 | 0.02 | 0.16 |  |  |  |  |  |  | STAT | DNAH1 | intronic |
| 3 | 52390555 | 0.93 | 0.98 | [rs66462821](http://www.broadinstitute.org/mammals/haploreg/detail_v2.php?query=&id=rs66462821) | C | T | 0.67 | 0.19 | 0.02 | 0.16 |  |  |  | Chorion,Fibrobl |  |  | 4 altered motifs | DNAH1 | intronic |
| 3 | 52391735 | 0.98 | 0.99 | [rs11714402](http://www.broadinstitute.org/mammals/haploreg/detail_v2.php?query=&id=rs11714402) | A | C | 0.3 | 0.17 | 0.02 | 0.16 |  |  |  | HMEC,iPS,Caco-2 |  |  | Sin3Ak-20,TATA | DNAH1 | synonymous |
| 3 | 52392877 | 0.94 | 0.99 | [rs11707864](http://www.broadinstitute.org/mammals/haploreg/detail_v2.php?query=&id=rs11707864) | G | A | 0.3 | 0.16 | 0.01 | 0.15 |  |  |  | Chorion,Medullo,pHTE |  |  | PLAG1,STAT | DNAH1 | intronic |
| 3 | 52399278 | 0.94 | 0.99 | [rs13075370](http://www.broadinstitute.org/mammals/haploreg/detail_v2.php?query=&id=rs13075370) | G | A | 0.29 | 0.15 | 0.01 | 0.15 |  |  |  | Th1,Fibrobl,pHTE |  |  | BDP1,Myc,RBP-Jkappa | DNAH1 | intronic |
| 3 | 52399627 | 0.94 | 0.99 | [rs11709658](http://www.broadinstitute.org/mammals/haploreg/detail_v2.php?query=&id=rs11709658) | G | A | 0.3 | 0.15 | 0.01 | 0.15 |  |  |  | Chorion |  |  |  | DNAH1 | intronic |
| 3 | 52400740 | 0.94 | 0.99 | [rs3752819](http://www.broadinstitute.org/mammals/haploreg/detail_v2.php?query=&id=rs3752819) | G | C | 0.3 | 0.15 | 0.01 | 0.15 |  |  |  | HSMM,HSMMtube |  |  |  | DNAH1 | intronic |
| 3 | 52403526 | 0.94 | 0.99 | [rs1077142](http://www.broadinstitute.org/mammals/haploreg/detail_v2.php?query=&id=rs1077142) | A | G | 0.66 | 0.19 | 0.02 | 0.16 |  |  |  |  |  |  | 13 altered motifs | DNAH1 | intronic |
| 3 | 52407933 | 0.98 | 0.99 | [rs13076890](http://www.broadinstitute.org/mammals/haploreg/detail_v2.php?query=&id=rs13076890) | T | C | 0.3 | 0.17 | 0.02 | 0.16 |  | H1 | K562, NHLF, GM12878 | 9 cell types |  |  | 6 altered motifs | DNAH1 | intronic |
| 3 | 52411929 | 0.95 | 0.99 | [rs67834575](http://www.broadinstitute.org/mammals/haploreg/detail_v2.php?query=&id=rs67834575) | T | A | 0.3 | 0.16 | 0.01 | 0.15 |  |  |  | Myometr |  |  | Sox,Zfp105 | DNAH1 | intronic |
| 3 | 52412006 | 0.95 | 0.99 | [rs67248391](http://www.broadinstitute.org/mammals/haploreg/detail_v2.php?query=&id=rs67248391) | A | T | 0.3 | 0.16 | 0.01 | 0.15 |  |  |  |  |  |  | Foxa,Foxd1 | DNAH1 | intronic |
| 3 | 52417638 | 0.94 | 0.99 | [rs28481563](http://www.broadinstitute.org/mammals/haploreg/detail_v2.php?query=&id=rs28481563) | T | C | 0.67 | 0.19 | 0.02 | 0.16 |  |  |  | Caco-2 |  |  | 12 altered motifs | DNAH1 | intronic |
| 3 | 52424001 | 0.94 | 0.99 | [rs9861879](http://www.broadinstitute.org/mammals/haploreg/detail_v2.php?query=&id=rs9861879) | A | G | 0.67 | 0.19 | 0.02 | 0.16 |  |  |  |  |  |  | HES1,Pax-4,ZEB1 | DNAH1 | intronic |
| 3 | 52431671 | 1 | 1 | [***rs390802***](http://www.broadinstitute.org/mammals/haploreg/detail_v2.php?query=&id=rs390802) | G | A | 0.21 | 0.15 | 0.02 | 0.16 |  |  |  | Gliobla |  |  | CAC-binding-protein,CCNT2,SP1 | DNAH1 | intronic |
| 3 | 52434967 | 0.95 | 1 | [rs498946](http://www.broadinstitute.org/mammals/haploreg/detail_v2.php?query=&id=rs498946) | T | C | 0.68 | 0.19 | 0.02 | 0.16 |  |  | K562 | HFF |  |  | CTCF,HES1 | 61bp 3' of BAP1 |  |
| 3 | 52442354 | 0.98 | 1 | [rs123602](http://www.broadinstitute.org/mammals/haploreg/detail_v2.php?query=&id=rs123602) | G | C | 0.31 | 0.16 | 0.02 | 0.15 |  | GM12878 | NHEK, HMEC |  |  |  | 7 altered motifs | BAP1 | intronic |
| 3 | 52471942 | 0.95 | -0.99 | [rs82825](http://www.broadinstitute.org/mammals/haploreg/detail_v2.php?query=&id=rs82825) | G | A | 0.69 | 0.84 | 0.98 | 0.85 |  |  | NHEK, HMEC | 8 cell types |  |  | 5 altered motifs | SEMA3G | intronic |
| 3 | 52477080 | 0.92 | -0.98 | [rs2016575](http://www.broadinstitute.org/mammals/haploreg/detail_v2.php?query=&id=rs2016575) | T | C | 0.69 | 0.84 | 0.98 | 0.85 |  |  |  |  |  |  |  | SEMA3G | intronic |
| 3 | 52482851 | 0.84 | -0.92 | [rs634382](http://www.broadinstitute.org/mammals/haploreg/detail_v2.php?query=&id=rs634382) | A | G | 0.34 | 0.81 | 0.98 | 0.84 |  |  | HepG2 |  |  |  | 7 altered motifs | 2.3kb 3' of TNNC1 |  |
| 3 | 52490391 | 0.86 | -0.93 | [rs34261027](http://www.broadinstitute.org/mammals/haploreg/detail_v2.php?query=&id=rs34261027) | A | G | 0.35 | 0.81 | 0.98 | 0.84 |  | 9 cell types |  | 17 cell types | ZNF263 |  |  | NISCH | intronic |
| 3 | 52501451 | 0.86 | -0.93 | [rs2159607](http://www.broadinstitute.org/mammals/haploreg/detail_v2.php?query=&id=rs2159607) | G | T | 0.7 | 0.83 | 0.98 | 0.84 |  |  |  |  |  |  | Ik-1 | NISCH | intronic |
| 3 | 52516293 | 0.88 | -0.95 | [rs6800707](http://www.broadinstitute.org/mammals/haploreg/detail_v2.php?query=&id=rs6800707) | C | G | 0.72 | 0.83 | 0.98 | 0.85 |  |  |  | 47 cell types | 4 bound proteins |  | HDAC2,Zfp161 | NISCH | intronic |
| 3 | 52523405 | 0.84 | -0.93 | [rs887515](http://www.broadinstitute.org/mammals/haploreg/detail_v2.php?query=&id=rs887515) | C | T | 0.33 | 0.8 | 0.98 | 0.84 |  |  | Huvec |  |  |  | 16 altered motifs | NISCH | missense |
| **rs12163565 and variants with r^2^ >= 0.8** | | | | |  |  |  |  |  |  |  |  |  |  |  |  |  |  |  |
| 3 | 52291078 | 0.8 | 0.91 | [rs57823393](http://www.broadinstitute.org/mammals/haploreg/detail_v2.php?query=&id=rs57823393) | G | A,C | 0.03 | 0.15 | 0.26 | 0.21 |  |  |  |  |  |  |  | WDR82 | 3'-UTR |
| 3 | 52430526 | 1 | 1 | [***rs12163565***](http://www.broadinstitute.org/mammals/haploreg/detail_v2.php?query=&id=rs12163565) | G | A | 0.03 | 0.14 | 0.25 | 0.2 |  |  |  |  |  |  | 4 altered motifs | DNAH1 | missense |
| 3 | 52488639 | 0.81 | 0.92 | [rs3821839](http://www.broadinstitute.org/mammals/haploreg/detail_v2.php?query=&id=rs3821839) | C | G | 0.02 | 0.14 | 0.26 | 0.21 |  | HepG2 | 7 cell types | 12 cell types |  |  | 4 altered motifs | 494bp 5' of NISCH |  |
| 3 | 52491046 | 0.81 | 0.92 | [rs2267921](http://www.broadinstitute.org/mammals/haploreg/detail_v2.php?query=&id=rs2267921) | T | C | 0.02 | 0.14 | 0.26 | 0.21 |  | 9 cell types |  | 6 cell types |  |  | En-1 | NISCH | intronic |
| 3 | 52491836 | 0.81 | 0.92 | [rs12629472](http://www.broadinstitute.org/mammals/haploreg/detail_v2.php?query=&id=rs12629472) | G | A | 0.02 | 0.14 | 0.26 | 0.21 |  | GM12878, NHEK | HMEC | 4 cell types |  |  | HES1,Myb | NISCH | intronic |
